# Direct resin composite restoration of maxillary central incisors using a 3D-printed template: two clinical cases

**DOI:** 10.1186/s12903-018-0621-4

**Published:** 2018-09-20

**Authors:** Juan Xia, Yinghua Li, Dongping Cai, Xilin Shi, Shiyong Zhao, Qianzhou Jiang, Xuechao Yang

**Affiliations:** 1 Department of Digital Dental Center, Stomatology Hospital of Guangzhou Medical University, Key Laboratory of Oral Medicine, Guangzhou Institute of Oral Disease, 59 Huangsha Road, Guangzhou, 510140 Guangdong Province China; 2 Department of Endodontics, Stomatology Hospital of Guangzhou Medical University, Key Laboratory of Oral Medicine, Guangzhou Institute of Oral Disease, 39 Huangsha Road, Guangzhou, Guangdong Province China

**Keywords:** 3D printing technology, Composite restoration, CAD/CAM, Fractured tooth, Dental caries

## Abstract

**Background:**

Three-dimensional (3D) printing technology is used widely in dentistry for applications including implant surgery, oral and maxillofacial surgery, orthognathic surgery, endodontics and prosthodontics. Using a 3D-printed template makes performing the repair procedure faster and more convenient. The aesthetic restoration of anterior teeth can recover facial beauty, enhance speaking and chewing functions and improve the quality of life of the patient.

**Case presentation:**

This article describes two kinds of clinical cases including fractured teeth and dental caries. In both, a 3D-printed template was used for direct resin composite restoration of maxillary central incisors. A 3D-printed template was built using the following 3-step process: data acquisition was conducted via intra-oral scanning, virtual modeling was performed using an imaging process, and manufacturing was performed using a 3D printer. Aesthetically restoring the maxillary incisors with the assistance of the 3D-printed template achieved the anticipated results, and the patients were very satisfied with the effect.

**Conclusions:**

The direct resin composite restoration of maxillary central incisors using a 3D-printed template represents a rapid, convenient, aesthetic and functional option for treating maxillary central incisors. A 3D-printed template is therefore an acceptable and reliable alternative to traditional direct composite restoration of maxillary central incisors including fractured teeth and dental caries.

## Background

The anterior teeth play an important role in facial beauty, and fully recovering a fractured anterior tooth requires restoration of the color, dental anatomy, and translucency of the tooth in addition to the curvature of the smile line and harmony with the other teeth in the arc [1]. The restored maxillary central incisors must also be well adapted, aesthetic, functional, and accepted by the patient.

Rapid prototyping technology, better known as 3-dimensional (3D) printing, is widely used for preoperative planning, procedure rehearsal and custom prosthetic design in clinical practice as well as an educational tool for teaching and to enhance communication between the patient and doctor [2, 3]. In dentistry, 3D printing technology is currently used in implant surgery [4, 5], oral and maxillofacial surgery [6, 7], orthognathic surgery [8, 9], prosthodontics [10, 11] and endodontics [12–14].

**Fig. 1:**
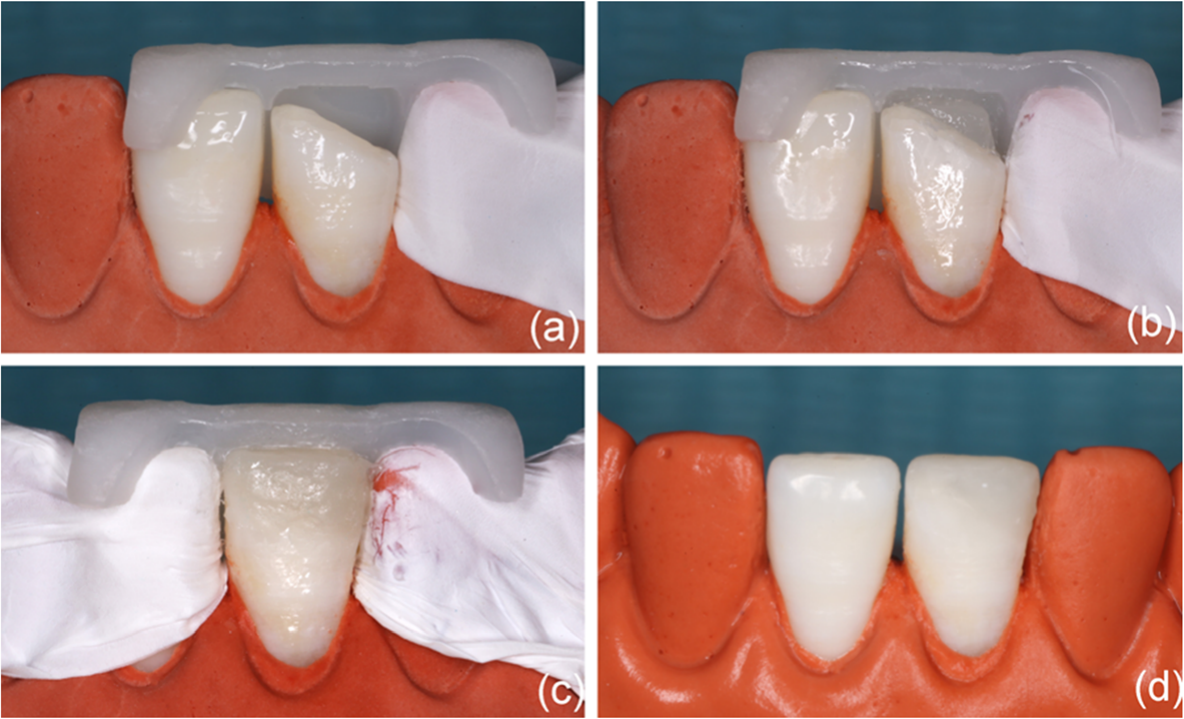
(**a**) The 3D printing template was positioned on plaster casts. (**b**) The palatal body was built up. (**c**) The restoration was finalized. (**d**) The final restoration was performed after polishing

Wong et al. [15] used 3D dental models and visualization techniques to analyze different parameters in smile arcs. Rosati et al. [16] used 3D morphological facial and dental analyses to aid practitioners during diagnosis and treatment planning. Weinlander et al. [17] introduced a new method for aesthetically evaluating the peri-implant mucogingival complex in which they used a collection of standardized oral photographs and computer-assisted measurements. However, to the best of our knowledge, no previous report has described the use of 3D printing technology for the aesthetic restoration of maxillary central incisors. Thus, we hypothesized that it would be possible to use 3D printing technology to aesthetically restore central incisors, and we performed a simulation experiment using a plaster model. First, we made a mandible plaster model and removed the middle third of the crown using a diamond bur. Second, we used a CEREC intra-oral scanner to digitally register the model and generation of the prosthesis using the fractured tooth. Third, we used Freeform to design the repair template and sent the stl-file to a 3D printer. Finally, with the aid of a 3D-printed template, we performed the restoration and achieved the anticipated results, including the appropriate color, dental anatomy and translucency of the tooth (Fig. 1).

## Case presentation

### Case 1

**Fig. 2:**
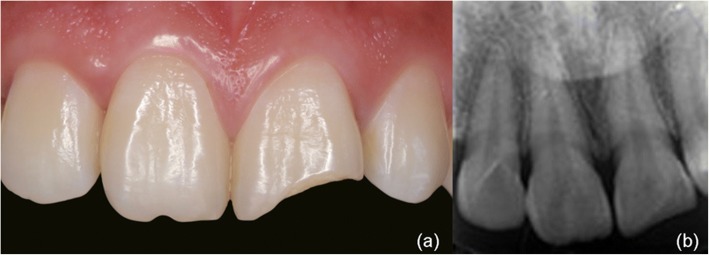
(**a**) Preoperative view of fractured maxillary anterior teeth. (**b**) Initial radiographic view of anterior teeth

A male patient, 26 years old, sought care at the dental clinic with fractures of the left maxillary central incisor resulting from a sudden strike three months earlier. The patient had no clinical symptoms during this period (Fig. 2a). A clinical examination revealed that the left maxillary central incisor was fractured in the middle third of the crown and that this fracture involved the enamel and dentin with no pulp exposure and no signs or symptoms of a concussion or contusion. A routine cold vitality test of the tooth revealed that it was associated with the same reaction as the reference tooth. Additionally, the patient had a defect in the incisal area of the right maxillary central incisor that resulted from eating melon seeds, and a routine cold vitality test of the tooth revealed a positive reaction. Finally, the relationship between the anterior teeth overbite and overjet was normal. A radiographic examination of the central incisors was conducted, and an analysis of radiography of the maxillary left central incisor revealed that there were fractures in the middle third of the crown, but no abnormalities, such as damage to the remaining roots, were observed (Fig. 2b).

**Fig. 3:**
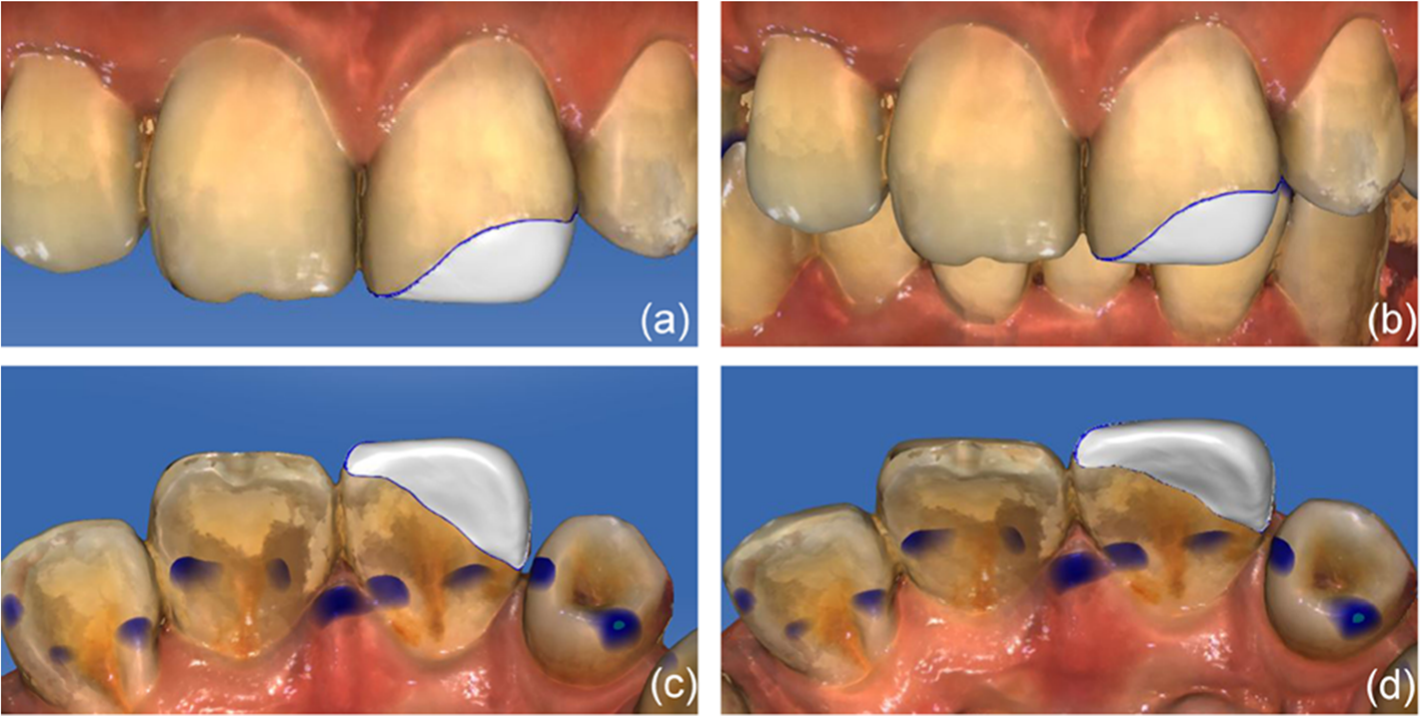
(**a**) Frontal view of the design of the prosthesis developed in CEREC AC. (**b**) Approximate aspect in occlusion. (**c**) Palatal view of the prosthesis. (**d**) Incisal view of the prosthesis

**Fig. 4:**
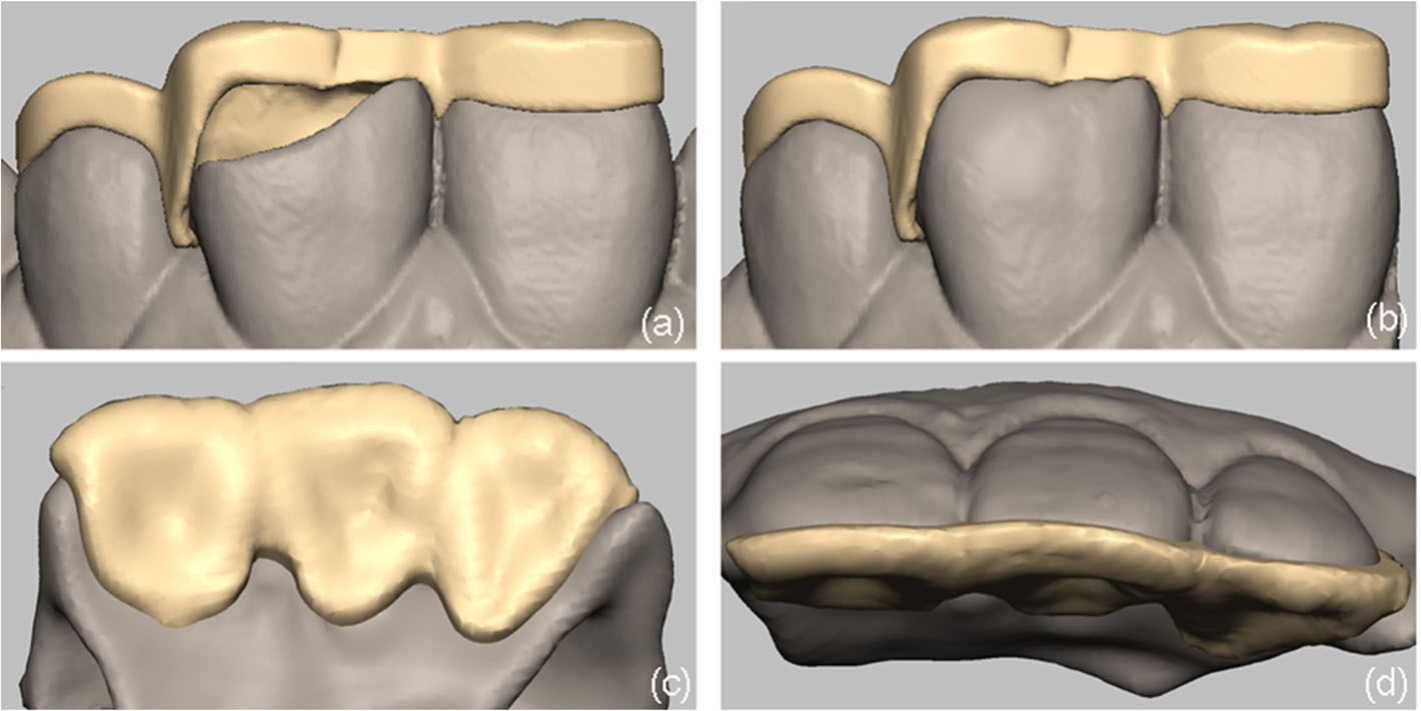
(**a**) The design used to repair the palatal guide without a prosthesis. (**b**) The repairing palatal guide in Freeform. (**c**) Palatal view of the repairing palatal guide. (**d**) Incisal view of the repairing palatal guide

**Fig. 5:**
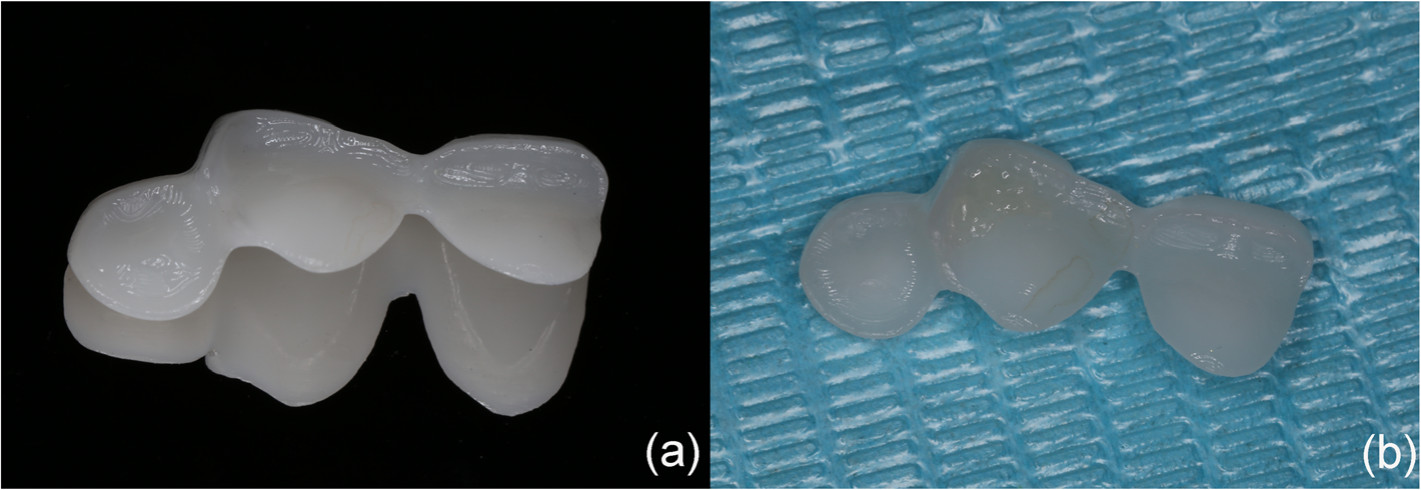
(**a**) The three-dimensional printed template in the mirror. (**b**) 3D printed template in the desk

A 3D-printed template was fabricated using intra-oral scanning, CAD, virtual modeling and 3D printing. Briefly, a digital registration of the dentition was performed using a CEREC AC Omnicam intra-oral scanner (CEREC AC D3492, Sirona Dental Systems GmbH, Fabrikstr, Bensheim, Germany). The inlay in the machine was selected, and the system automatically generated a prosthesis using the contralateral tooth as a reference. From the analysis performed using the software, the occlusal contact of the intercuspal occlusion of the patient was concentrated in the middle third of the cervix, and it was therefore appropriate for composite resin restoration. An occlusal adjustment was made to eliminate anterior contact in the occlusion and to avoid contact with the prosthesis (Fig. 3). We showed a picture of the result to the patient, and he was satisfied with it. The data were then imported into Freeform (Geomagic Freeform, 3D Systems, Morrisville, North Carolina, USA), a software program that is widely used to design 3D models. Using the Freeform program, a template can be designed through a process similar to drawing a picture, and a dentist can design a repaired palatal template in only minutes (Fig. 4). The digitally designed template is prepared for export using the “stl check” command, exported as a stl-file and then sent to a 3D printer (3D System 3510HB, 3D Systems, Morrisville, North Carolina, USA). Finally, the 3D-printed template is fabricated (Fig. 5).

**Fig. 6:**
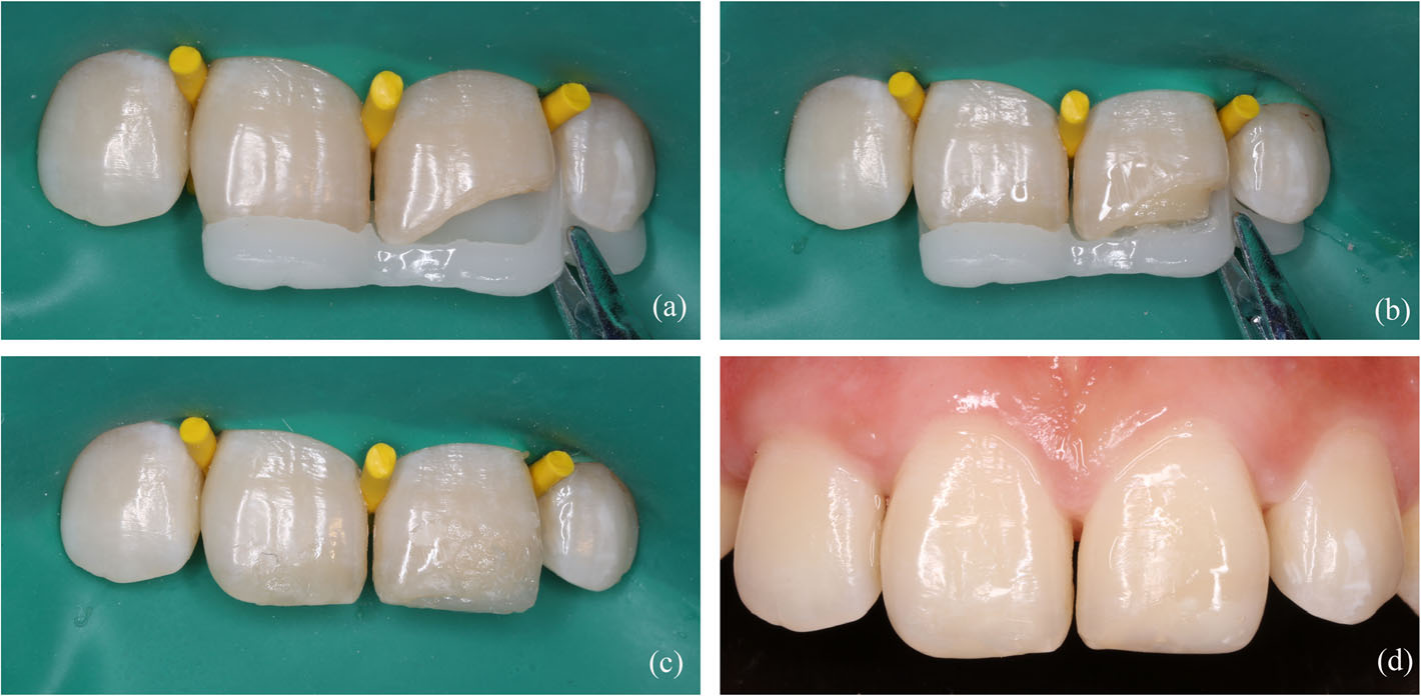
(**a**) The 3D printing template was placed on the anterior teeth. (**b**) The palatal surface and dentin core were augmented. (**c**) The restoration was completed before polishing. (**d**) The teeth were finished and polished

Before treatment, the 3D-printed template was detached and soaked in disinfectant. Then, the template was positioned on the patient’s dentition, and a correct and reproducible fit was verified. Initially, the anterior teeth were isolated using a rubber dam (Hygienic Elasti rubber dam, Switzerland). The teeth were carefully cleaned using prophylaxis paste (SS white prophylaxis paste, England), dried, and submitted to minimal tooth preparation using a diamond bur (Mani SF-41, Japan) to produce an improved alignment for the bond. Both surfaces of the connection were etched using acid gel (Ultra-Etch® 35% Phosphoric Acid, Ultradent, USA), rinsed, and gently dried. Single bond (Adper™ Single Bond 2, 3 M ESPE, USA) was applied first. The surface was then air-dried for 5 s and exposed to light-activation for 10 s. Subsequently, the 3D template, which had been detached and soaked in disinfectant, was positioned on the back of the anterior teeth (Fig. 6a). It was convenient to construct the palatal surface using an opaque enamel shade (E2, Ceram*X duo, DENTSPLY, Germany) with the aid of a 3D printing guide. After polymerization, the palatal wall is sufficiently strong to support the next stratification steps. Reconstruction was performed using an opaque dentin shade (D2, Ceram*X duo, DENTSPLY, Germany) to construct the dentin body (Fig. 6b). The enamel shade E2 was used to match the superficial enamel, and each composite increment was light-cured for 20 s. Additionally, tooth 11 was restored using enamel shade E2 in the incisal area and on the buccal surface. The final step consisted of performing an additional 20 s of polymerization at each site. After excess composite material was removed, an occlusion test was performed using carbon paper, and the restorations were shaped to a proper anatomic morphology (Fig. 6c). Next, finishing and polishing procedures were performed using diamond fine coating burs and a polishing system (One-step diamond micro-polisher, Germany) (Fig. 6d).

### Case 2

**Fig. 7:**
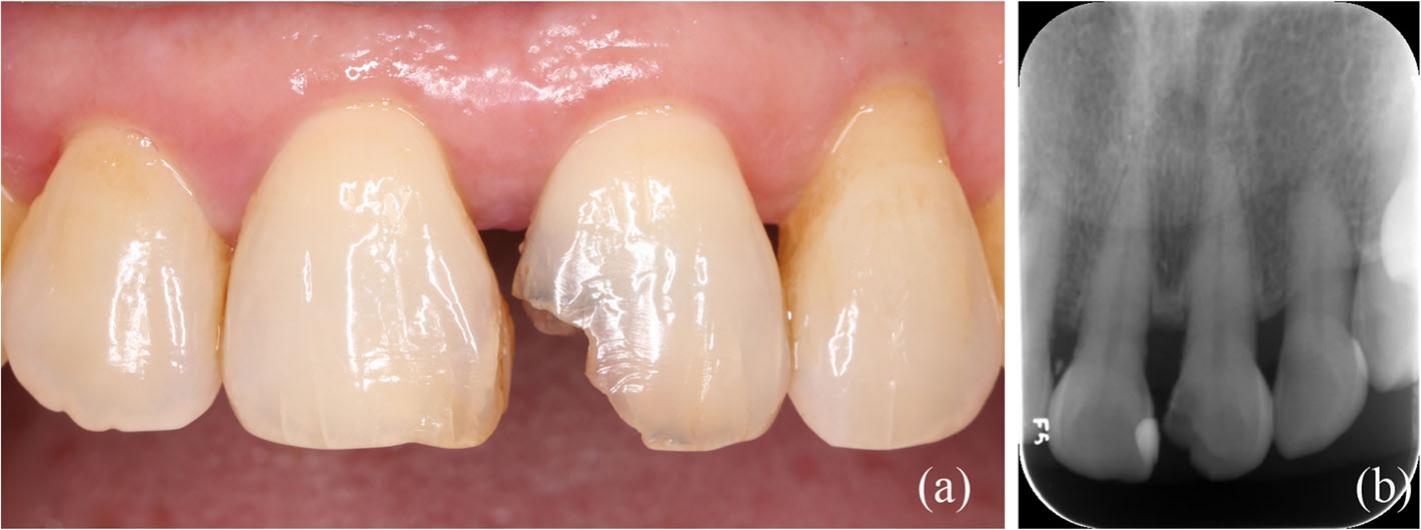
(**a**) Preoperative view of dental caried maxillary anterior tooth. (**b**) Initial radiographic view of anterior teeth

**Fig. 8:**
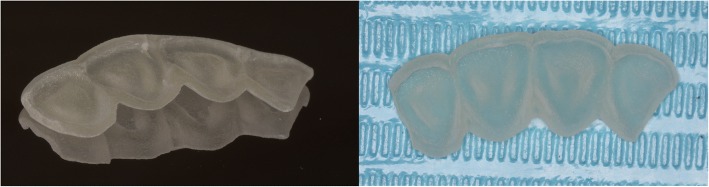
The three-dimensional printed template

A 61-year-old female patient was referred to the clinic with dental caries of her left maxillary central incisor. The patient had no clinical symptoms (Fig. 7a). A clinical examination revealed that the left maxillary central incisor had caries in the middle third of the crown, which involved the enamel and dentin with no pulp exposure. A routine cold vitality test revealed that the tooth was sensitive. Finally, the relationship between the anterior teeth overbite and overjet was normal. A radiographic examination of the central incisors was conducted, and a radiographic analysis of the maxillary left central incisor revealed that there were caries in the middle third of the crown. (Fig. 7b). A 3D-printed template was fabricated using intra-oral scanning, CAD, virtual modeling and 3D printing as in the first case. Finally, the 3D-printed template was fabricated (Fig. 8).

**Fig. 9:**
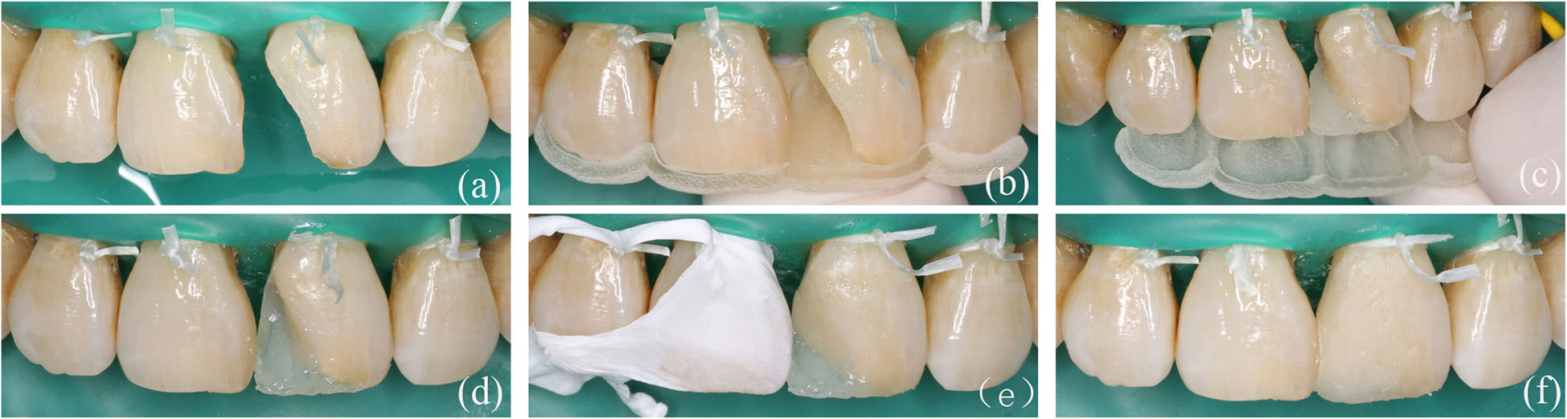
(**a**) Functional esthetic bevel (**b**) The 3D printing template was placed on the anterior teeth. (**c**) The palatal surface were augmented with template (**d**) The palatal surface was constructed after polishing (**e**) The dentin core (**f**) The restoration was completed before polishing

**Fig. 10:**
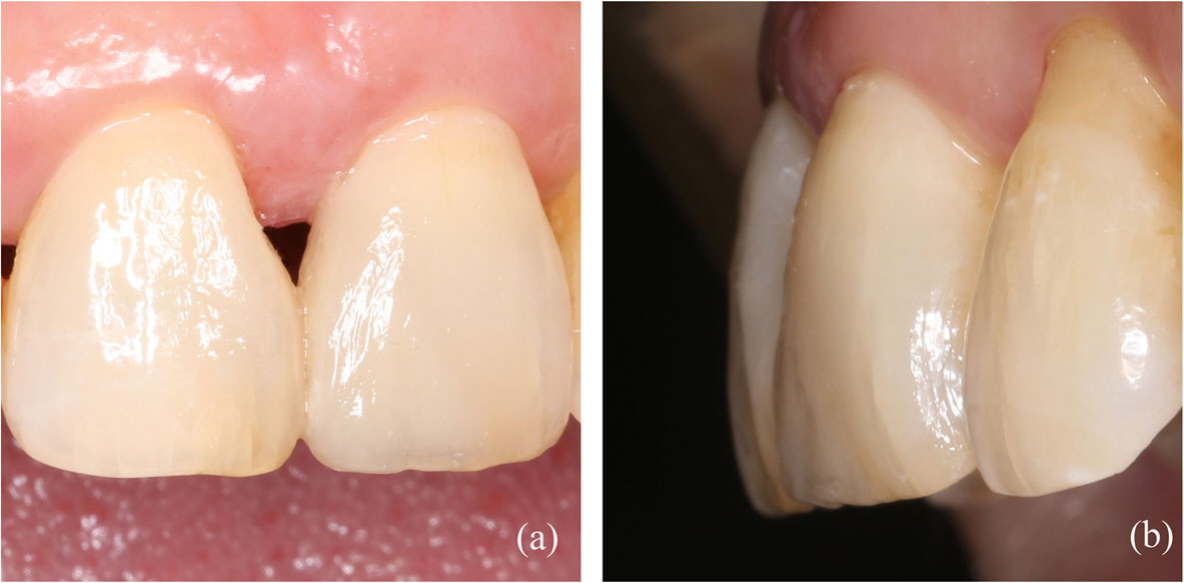
(**a**) The labial surface of the completed case. (**b**) Lateral view of the teeth

Before treatment, the 3D-printed template was detached and soaked in disinfectant. Then, the template was positioned on the patient’s dentition, and a correct and reproducible fit was verified. Initially, the anterior teeth were isolated using a rubber dam. The teeth were subjected to minimal tooth preparation using a diamond bur (Mani SF-41, Japan) to produce an improved alignment for the bond (Fig. 9a). Both surfaces of the connection were etched using acid gel (Ultra-Etch® 35% Phosphoric Acid, Ultradent, USA), rinsed, and gently dried. Single bond (Adper™ Single Bond 2, 3 M ESPE, USA) was applied first. The surface was then air-dried for 5 s and exposed to light activation for 10 s before the appropriate enamel composite (E3, Ceram*X duo, DENTSPLY, Germany) was placed on the defect area of the 3D template. Subsequently, the 3D template was positioned on the back of the anterior teeth (Fig. 9b) and exposed to light activation for 20 s (Fig. 9c). The palatal surface was then constructed. After polymerization, the palatal wall was sufficiently strong to support the next stratification steps (Fig. 9d). The integration of A2 (Ceram*X duo, DENTSPLY, Germany) was used to match the functional aesthetic bevel. Reconstruction was performed using an opaque dentin shade (D2, Ceram*X duo, DENTSPLY, Germany) to construct the dentin body (Fig. 9e). The enamel shade E3 was used to match the superficial enamel, and each composite increment was light cured for 20 s. The final step consisted of performing an additional 20 s of polymerization at each site. After excess composite material was removed, an occlusion test was performed using carbon paper, and the restorations were shaped to the proper anatomic morphology (Fig. 9f). Next, finishing and polishing procedures were performed using fine diamond-coated burs and a polishing system (One-step diamond micro-polisher, DENTSPLY, Germany). Figure 10 shows the final appearance of the restorations as follows: labial view (Fig. 10a) and lateral view (Fig. 10b).

## Discussion

Digital dentistry can be broadly defined as any dental technology or device that incorporates digital or computer-controlled components, and it is changing the shape of the dental industry. The digital dentistry revolution has begun. In this study, the authors discussed the advantages and disadvantages of digital dentistry. The main advantages are as follows: first, digital dentistry is a powerful treatment planning tool that has improved the efficiency of diagnosis and treatment; second, it provides a high level of predictability in outcomes and enables good communication among dental team members while also improving accuracy over previous methods; and third, it promotes patient education and treatment acceptance. Its disadvantages include the following: first, the costs associated with equipment, maintenance and medical expenses and second, a lack of adequately trained clinicians and teams because it is a new dental technology [18]. In conclusion, the digital revolution has opened interesting concepts and possibilities, but it also represents a challenge for dentists. Therefore, it is necessary to learn about new knowledge, including new devices, software and machines [19].

Because aesthetics are based on subjective and individual differences, it is important for a preoperative prediction of an aesthetic effect to reflect good communication between a doctor and patient. Moreover, such discussions are of great significance when deciding whether to proceed with a repair and when setting expectations to prevent future disputes. Currently, in clinical aesthetic restorations, diagnostic or temporary restoration methods are often used to predict the aesthetic effect [20]. Diagnostic wax is commonly used in clinics, but its use is limited to simulating tooth size and shape, and it is therefore difficult to imagine the visual effect of the prosthesis in the mouth [21]. In the present article, the aesthetic restoration of a fractured tooth was achieved by performing 3D scanning of the dentition and using a CAD system and 3D design software. CAD can be used to model the desired form and has the potential to further improve the quality of the patient’s smile. The young clinician can discuss the planning with a more experienced doctor at the computer before calling the patient for a second appointment. We believe that proper planning is key for success in all disciplines of dentistry. Although making a 3D-printed template requires more time for preparation and higher cost, this procedure could certainly be suitable for young and unexperienced doctors who have no experience in correctly reconstructing the form or shape of a central incisor; the reconstruction of the form and shape is key to aesthetics. However, making a 3D-printed template could be considered a waste of time for the experienced clinician, who can restore these teeth directly without any physical template, with regard to the time spent in the planning. Furthermore, digital dentistry is a powerful treatment planning tool; patients can intuitively feel the restored teeth, and patients may feel more comfortable and relaxed in the clinic. Currently, direct reconstructions performed using a silicone guide can be performed in cases involving crown fractures, inadequate fillings caries, closing diastema, or wear lesions. This type of restoration involves a minimally invasive therapy intervention, and a silicone build-up guide is frequently used during the aesthetic management of a tooth with direct composite. A 3D-printed template can perform all of the above functions. Digital technologies allow us to accurately analyze and evaluate occlusions to make an appropriate treatment plan. Compared to traditional direct composite restorations, direct resin composite restoration with the aid of a 3D-printed template necessitates a new machine and more time for preparation, but it saves time in the dentist’s chair. Although the cost is not lower, with the aid of a 3D-printed template, the doctor could improve the efficiency and aesthetic effects in the clinic, and the patient would feel more comfortable and have good communication with the doctor. In contrast to a silicone guide, a 3D-printed template does not require a silicone rubber impression of the patient to be made, and the patients will be more comfortable and have a more pleasant experience while sitting in the dentist’s chair. This is especially important for patients who are sensitive to silicone rubber. Additionally, a 3D-printed template does not require laboratory processing and can be generated in a dental clinic. Finally, using this technique does not require many of the traditional production processes that are currently performed during the repair process, including a dental impression, perfusion model, carving prosthesis wax type and the embedding casting process. This enables substantial savings with regard to resources and avoiding environmental pollution.

Techniques involving 3D printing have initiated a new age in dentistry. These techniques have already changed dentistry and will increasingly replace a number of traditional techniques involved in fabricating dental restorations. The limitations of 3D printing include its cost and complexity and the fact that it is time-consuming. Although 3D printers are becoming more affordable, the costs associated with operating a 3D machine, obtaining the required materials, maintaining the equipment, and training skilled operators must be carefully considered [22]. Generally, medical applications involving 3D printing show promise for promoting specialized surgical planning and prosthetics applications [23].

In conclusion, the costs associated with equipment, maintenance and medical expenses should be considered. This new technology will take more time for preparation and more spending, and there is a lack of adequately trained clinicians and teams. Thus, it is necessary for clinicians to learn about new areas of knowledge, including new devices, software and machines.

## Conclusions

The aim of this article is to describe an uncomplicated approach to using a 3D-printed template and resin composites to restore and enhance the aesthetic appearance of the anterior dentition. With the help of digital technology, the patient could feel more comfortable and relaxed in the clinic. Young and unexperienced doctors could improve their efficiency and quality in the clinic. The direct resin composite restoration of maxillary central incisors using a 3D-printed template represents a rapid, convenient, aesthetic and functional option for the direct resin composite restoration of maxillary central incisors. A 3D-printed template is therefore an acceptable and reliable alternative to traditional direct composite restoration of maxillary central incisors including fractured teeth and dental caries.
